# The Cell and Molecular Biology of Neurodegenerative Diseases: An Overview

**DOI:** 10.3389/fneur.2013.00194

**Published:** 2013-11-29

**Authors:** Heather L. Montie, Thomas M. Durcan

**Affiliations:** ^1^Department of Bio-Medical Sciences, Philadelphia College of Osteopathic Medicine, Philadelphia, PA, USA; ^2^Montreal Neurological Institute, McGill University, Montreal, QC, Canada

**Keywords:** neurodegeneration, aggregation, polyglutamine, Parkinson’s disease, Alzheimer disease, ALS

In this research topic, the primary focus is on understanding the cellular and molecular mechanisms in the pathogenesis of different neurodegenerative disorders. These include Alzheimer’s disease (AD), Parkinson’s disease (PD), and polyglutamine (polyQ) expansion diseases. To date, no cure exists for these disorders and it is paramount that research efforts continue to focus on understanding the molecular underpinnings behind these disorders. This will enable better symptom-directed therapeutics and perhaps even curative treatments to be developed.

Throughout this topic, it becomes evident that there are common cellular pathways that are altered in these disorders, including protein, mitochondrial, and transcriptional homeostasis. In the case of AD, it has become widely accepted that AD is a synaptopathy, meaning that there is a loss or damage of synapses. This damage to synapses leads to altered neuronal circuitry. The neuron-specific, post-synaptic protein, Arc, has gained recent attention for its contribution in the regulation of memory consolidation. Kerrigan and Randall (1) discuss how alterations of Arc protein in the brains of AD patients and animal models of AD may be a clue as to how synaptic transmission is altered in AD, and how this cellular pathway may be of interest for therapeutic development.

The next three reviews discuss the molecular events underlying PD and how the normal function of specific proteins associated with PD can help shed light on the causes of familial and sporadic PD. Lim and Zhang (2) outline a range of studies that implicate aberrations in mitochondrial function and protein homeostasis, with oxidative stress as the possible link between these two. A review from Dr. Edward A. Fon’s group complements this discussion by focusing on the structure and function of Parkin, PINK1, and DJ-1 as they relate to PD (3). The second review from Dr. Fon’s group digs even deeper into the role of Parkin and PINK1 in mitophagy in neurons. They discuss the importance of research initiatives to better define the roles of these two proteins in mitophagy and, in particular, within the context of a neuronal setting (4).

The next five reviews focus on polyQ expansion diseases. Almeida et al. (5) provide a structural and functional view of trinucleotide repeats and encoded homopeptide expansions, emphasizing polyQ expansions and their role in inducing the self-assembly, aggregation, and functional alterations of the protein, leading to neuronal toxicity and cell death. These authors focus on ataxin-3 and huntingtin (Htt), the main protein implicated in Machado–Joseph Disease (MJD) and Huntington’s disease (HD) respectively. Drs. Durcan and Fon, also focus on ataxin-3 and its function as a deubiquitinating enzyme (6). These authors have recently identified ataxin-3’s E3 ubiquitin ligase partners to be parkin and CHIP. As MJD patients often present with PD symptoms, the fact that parkin’s activity is regulated by ataxin-3-mediated deubiquitination is a critical link for this phenotype. As mutant, but not wild-type ataxin-3 promotes clearance of parkin via the autophagy pathway, there seems to be a possibility that increased turnover of parkin contributes to pathogenesis in MJD. Moreover, ataxin-3 also induces a reduction in CHIP levels. In light of these findings, the authors discuss the implications for the role of mutant ataxin-3’s effect upon Parkin and CHIP levels in understanding the molecular processes involved in SCA3 and perhaps other neurodegenerative disorders.

Moumné et al. (7) discuss the role of transcriptional disruption in HD, specifically, as it relates to the transcriptional repressor, R element-1 silencing transcription factor (REST). REST is a transcriptional repressor of neuronal survival factors and normally associates with wild-type Htt. However, there is an aberrant alteration of cytoplasmic retention of the transcriptional repressor REST by mutant Htt. The authors go on to describe studies that have implicated this aberrant effect of mutant Htt on REST function to regulate neuronal genes and how this may impact pathogenesis in HD. Du et al. (8) focus on depression, the major psychiatric manifestation of HD. They discuss potential mechanisms of pathogenesis identified from animal models and compare depression in HD patients with non-HD persons, asking the question; Does HD-related depression differ from non-HD persons? They also go on to discuss some molecular and cellular mechanisms which may contribute to depression in HD.

Beitel et al. (9) discuss spinal and bulbar muscular atrophy (SBMA), a polyQ expansion disease caused by the expansion of a CAG tract in the androgen receptor (AR) gene. This review summarizes all of the aspects of AR metabolism, from posttranslational modifications, to protein degradation and transcriptional function that have been implicated in SBMA pathogenesis.

Spencer et al. (10) offer a commentary on Western Pacific Amyotrophic lateral sclerosis (ALS) – parkinsonism-dementia complex (PDC) that plagues the island populations of Chamorros on Guam, Japanese in Honshu Island’s Kii Peninsula, and Papuan New Guineans in Irian Jaya, Indonesia. It is a spectrum disorder believed to be to be triggered by a toxin in the seed of the neurotoxic cycad plant. This toxin is thought to induce a prototypical neurodegenerative disorder linked to DNA damage and aberrant proteogenesis.

The final review discusses how cellular surfaces modulate protein aggregation related to neurodegeneration. The interaction of proteins with liquid/surface interfaces is a fundamental phenomenon with potential implications for protein-misfolding diseases. Burke et al. (11) provide an overview of what is known about the influence of (sub) cellular surfaces in driving protein aggregation and/or stabilizing specific aggregate forms and how further understanding of such could provide new insights into toxic mechanisms associated with these diseases.

Lastly, the methods paper by Lange et al. (12) describes a detailed method for culturing embryonic dorsal root ganglion neurons for Seahorse Extracellular Flux XF24 analysis. This is a procedure used to measure the relative state of glycolytic and aerobic metabolism in live cells, in order to assess mitochondrial function. As changes in mitochondrial dynamics and function contribute to multiple neurodegenerative diseases, this method outlined herein is of significant interest to this topic.

## References

[1] Kerrigan TL Randall AD A new player in the “synaptopathy” of Alzheimer’s disease – Arc/Arg 3.1. Front Neur (2013) 4:9 10.3389/fneur.2013.00009 23407382 PMC3570765

[2] Lim K-L Zhang CW Molecular events underlying Parkinson’s disease – an interwoven tapestry. Front Neurol (2013) 4:33 10.3389/fneur.2013.00033 23580245 PMC3619247

[3] Trempe J-F Fon EA Structure and function of Parkin, PINK1, and DJ-1, the three musketeers of neuroprotection. Front Neurol (2013) 4:38 10.3389/fneur.2013.00038 23626584 PMC3630392

[4] Grenier K McLelland G-L Fon EA Parkin- and PINK1-dependent mitophagy in neurons: will the real pathway please stand up? Front Neurol (2013) 4:100 10.3389/fneur.2013.00100 23882257 PMC3715719

[5] Almeida B Fernandes S Abreu IA Macedo-Ribeiro S Trinucleotide repeats: a structural perspective. Front Neurol (2013) 4:76 10.3389/fneur.2013.00076 23801983 PMC3687200

[6] Durcan TM Fon EA Ataxin-3 and its E3 partners: implications for Machado–Joseph disease. Front Neurol (2013) 4:46 10.3389/fneur.2013.00046 23653622 PMC3644722

[7] Moumné L Betuing S Caboche J Multiple aspects of gene dysregulation in Huntington’s disease. Front Neurol (2013) 4:127 10.3389/fneur.2013.00127 24167500 PMC3806340

[8] Du X Pang TYC Hannan AJ A tale of two maladies? Pathogenesis of depression with and without the Huntington’s disease gene mutation. Front Neurol (2013) 4:81 10.3389/fneur.2013.00081 23847583 PMC3705171

[9] Beitel LK Alvarado C Mokhtar S Paliouras M Trifiro M Mechanisms mediating spinal and bulbar muscular atrophy: investigations into polyglutamine-expanded androgen receptor function and dysfunction. Front Neurol (2013) 4:53 10.3389/fneur.2013.00053 23720649 PMC3654311

[10] Spencer PS Fry RC Palmer VS Kisby GE Western Pacific ALS-PDC: a prototypical neurodegenerative disorder linked to DNA damage and aberrant proteogenesis? Front Neur (2012) 3:180 10.3389/fneur.2012.00180 PMC3527830 23267344

[11] Burke KA Yates EA Legleiter J Biophysical insights into how surfaces, including lipid membranes, modulate protein aggregation related to neurodegeneration. Front Neurol (2013) 4:17 10.3389/fneur.2013.00017 23459674 PMC3585431

[12] Lange M Zeng Y Knight A Windebank A Trushina E Comprehensive method for culturing embryonic dorsal root ganglion neurons for Seahorse Extracellular Flux XF24 analysis. Front Neur (2012) 3:175 10.3389/fneur.2012.00175 23248613 PMC3522103

